# Discovery of novel anti-*Prototheca* compounds with diverse modes of action through screening of a chemical library

**DOI:** 10.1128/msphere.00427-26

**Published:** 2026-10-07

**Authors:** Amir Aliramezani, Magdalena Widziolek, Łukasz Chajec, Gabriela A. Żyłka, Beatrycze Nowicka, Izabela Ciastoń, Patrizia Danesi, Tomasz Jagielski, Magdalena Rost-Roszkowska, Tomasz K. Prajsnar, Jakub M. Kwiecinski

**Affiliations:** 1 Department of Microbiology, Faculty of Biochemistry, Biophysics and Biotechnology, Jagiellonian University 37799, Kraków, Poland; 2 Department of Evolutionary Immunology, Institute of Zoology and Biomedical Research, Faculty of Biology, Jagiellonian University, Kraków, Poland; 3 Institute of Biology, Biotechnology and Environmental Protection, University of Silesia in Katowice 564786, Katowice, Poland; 4 Chair of Epidemiology and Preventive Medicine, Faculty of Medicine, Jagiellonian University Medical College 49573, Kraków, Poland; 5 Department of Plant Physiology and Biochemistry, Faculty of Biochemistry, Biophysics and Biotechnology, Jagiellonian University 37799, Kraków, Poland; 6 Istituto Zooprofilattico Sperimentale delle Venezie 83372, Legnaro, Padua, Italy; 7 Department of Medical Microbiology, Institute of Microbiology, Faculty of Biology, University of Warsaw 49605, Warsaw, Poland; Kyungpook National University, Daegu, Republic of Korea

**Keywords:** *Prototheca*, antimicrobial agents, antifungal agents, drug discovery, drug repurposing, drug screening

## Abstract

*Prototheca*, a genus of unpigmented yeast-like microscopic algae, is an emerging pathogen of humans and animals. Treatment of *Prototheca* infections is often unsuccessful because the unusual algal biology of these pathogens limits the availability of effective therapeutics. Here, we screened the Pandemic Response Box, a library of 400 compounds with potential anti-infective activity that are either clinically approved or in preclinical development, and identified five promising compounds with anti-*Prototheca* activity: tafenoquine, OSU-03012, alexidine, ciclopirox, and eberconazole. Characterized alongside amphotericin B, which is currently often used for treatment, these identified compounds displayed potent activity against multiple *Prototheca* strains and species by inhibiting growth, killing the algae, and disrupting biofilms. Their diverse mechanisms of action included effects on cell walls and membranes, mitochondrial dysfunction, ROS overproduction, metabolic alterations, and induction of apoptosis. Most of the identified compounds were also effective against *in vivo Prototheca* infection in larval zebrafish, highlighting their translational potential and the possibility of future introduction into clinical practice.

**IMPORTANCE:**

This study addresses the emerging threat of infections caused by *Prototheca*, a group of unusual algae that can cause difficult-to-treat infections in both humans and animals. Current treatment options are limited and often ineffective, highlighting the need to develop new therapeutic strategies, especially as reported cases of *Prototheca* infections quickly increase. Here, we identified several compounds with strong activity against *Prototheca*. These agents not only blocked growth but also killed the microorganisms, damaged their biofilms, and reduced infection in an animal model. Importantly, many of the identified compounds are already used clinically for other indications, which raises the possibility of rapid repurposing against *Prototheca*. Moreover, we identified multiple mechanisms responsible for these compounds’ activity, providing a framework for the future development of even more potent anti-*Prototheca* therapies.

## INTRODUCTION

Yeast-like microorganisms from the *Prototheca* genus are emerging pathogens of humans and animals (1, 2). Despite their resemblance to fungi, *Prototheca* are unicellular, unpigmented Chlorophyta algae—which makes them a unique group of microbial pathogens (3). *Prototheca* are mainly responsible for bovine mastitis (with infection prevalence of about 10% of cows in affected herds, and up to even half of the herds being affected), but infections in humans, dogs, and other animals are quickly increasing worldwide (1, 4).

The species most often associated with disease are *Prototheca wickerhamii* (in humans and dogs) and *Prototheca bovis* (in humans, dogs, and cattle). Less commonly, infections can be caused by *Prototheca blaschkeae*, *Prototheca ciferrii*, *Prototheca cutis*, and *Prototheca miyajii* (2). Protothecosis in otherwise healthy humans usually manifests as a localized (often skin) infection, while immunosuppressed individuals suffer from disseminated and bloodstream infections (1, 2). In dogs, the disease most often manifests as a disseminated infection with colitis as its focal point (4, 5). Importantly, *Prototheca* infections are characterized by an insidious onset, aggressive course, limited response to therapy, and high mortality. Even when treated, the mortality in disseminated cases exceeds 50% (in humans), or even 90% (in dogs) (1, 2, 5).

Very little is known about the pathogenesis of *Prototheca* infections, which complicates their treatment (6). An even greater therapeutic challenge in protothecosis is the lack of dedicated drugs. *Prototheca* are usually susceptible to the antifungal amphotericin B (AMB), which is therefore often used in treatment (especially in systemic infections), and many strains also respond to itraconazole (ITC), fluconazole, or other azole drugs (2, 7–9). Other antifungal or antibacterial agents are also occasionally used (2), but the overall anti-*Prototheca* repertoire remains extremely limited. Notably, the combined use of two or more drugs from different classes appears to improve infection outcomes (5); however, the scarcity of available agents limits this approach. Collectively, these factors highlight the urgent need to identify and develop new anti-*Prototheca* compounds with diverse mechanisms of action. This effort is complicated by *Prototheca* being algae, and therefore differing in their biology from other pathogenic microbes for which anti-infective drugs are typically designed.

Various approaches have been employed for discovering anti-*Prototheca* drugs. These include testing already existing antibiotics and antifungals (7–11). Compounds with broad nonspecific antimicrobial activity, such as disinfectants (12, 13), essential oils (11, 14), antimicrobial peptides (15), antimetabolites (16), and silver nanoparticles (17, 18), have also been evaluated. Finally, anti-plant compounds and herbicides have also been considered (19, 20). All these approaches, however, are limited by their focus on a small pool of already known structures and mechanisms of action. An alternative approach proposed here is the screening of large compound libraries. This could not only identify structurally novel anti-*Prototheca* compounds, but also reveal unexpected mechanisms of action for future investigation. Moreover, screening libraries of compounds already undergoing clinical development could accelerate the process of bringing the identified agents to market and into clinical practice (21).

A perfect possibility for such large-scale anti-*Prototheca* screening is provided by the “Pandemic Response Box” chemical library created by the Medicines for Malaria Venture. It contains 400 structurally diverse compounds with expected antibacterial, antiviral, or antifungal activity, already available on the market or in various phases of drug discovery and development (22). In this study, by screening the Pandemic Response Box, we successfully identified five novel anti-*Prototheca* compounds, characterized their efficacy *in vitro* and *in vivo*, and identified their mechanisms of action, revealing novel therapeutic targets for protothecosis.

## RESULTS

### *In vitro* screening identifies compounds with anti-*Prototheca* activity

**Fig 1:**
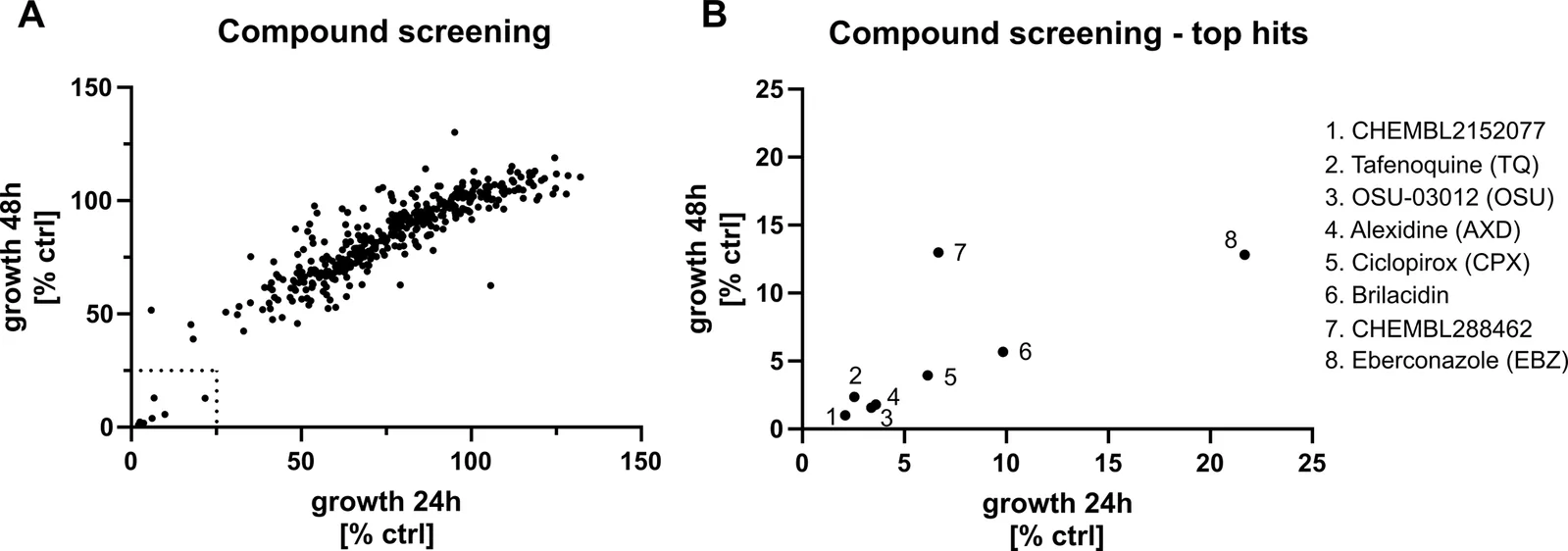
Screening of the “Pandemic Response Box” chemical library for anti-*Prototheca* activity against *P. wickerhamii* CCUG 50031. (**A**) Efficacy of 400 compounds was assessed based on the growth inhibition (% of control growth) at 24 h and 48 h time points. (**B**) The magnified lower quadrant of the screen with the eight most inhibitory hits. Data are shown as means; *n* = 2.

An initial screen of 400 compounds from the Pandemic Response Box identified eight candidates capable of reducing the growth of *P. wickerhamii* CCUG 50031 to 25% or less of the untreated control, after both 24 and 48 h of incubation (Fig. 1A and B; Data S1). In order from the most effective to the least, these were CHEMBL2152077 compound, tafenoquine (TQ), OSU-03012 (OSU), alexidine dihydrochloride (AXD), ciclopirox (CPX), brilacidin, CHEMBL288462 compound, and eberconazole (EBZ).

The two CHEMBL compounds, synthesized as potential antibacterials over a decade ago (23, 24), and brilacidin, a wide-spectrum antimicrobial peptide mimetic (25), were unavailable for subsequent experiments at the time of screening completion. Therefore, the five remaining hits were selected for detailed analysis: TQ, OSU, AXD, CPX, and EBZ. AMB was also included in all subsequent experiments, as it is currently often used for *Prototheca* treatment.

The selected compounds were tested against a panel of *Prototheca* strains and species isolated from infected humans, dogs, and other animals. All compounds showed broad efficacy, as evident in their minimal inhibitory concentrations (MICs) and minimal cidal concentrations (MCCs) (Table 1). AXD was highly active against all tested strains at concentrations comparable to those of AMB. CPX displayed similarly strong growth-inhibitory activity but lacked consistent cidal activity. TQ and OSU were also active against all strains, although at higher concentrations than AMB. In the case of EBZ, complete growth inhibition required relatively high concentrations, consistent with the characteristic minimal “trailing growth” commonly observed for higher concentrations of azole antifungals. However, when concentrations producing 50% growth inhibition were analyzed (as is recommended for azole susceptibility testing), EBZ showed strong activity against most strains.

**TABLE 1:** MIC and MCC values for compounds tested against a range of microorganisms^*a*^

Organism	AMB		TQ		OSU		AXD		CPX		EBZ		
	MIC	MCC	MIC	MCC	MIC	MCC	MIC	MCC	MIC	MCC	MIC	MCC	50% inhibition
*Prototheca*													
*P. wickerhamii*													
CCUG 50031	1	1	8	8	2	4	1	1	1	1	16	16	0.125
PWPL1	0.5	2	4	4	4	8	1	1	0.5	>32	8	8	<0.06
WP5	1	8	4	8	4	16	0.5	1	1	>32	16	16	<0.06
*P. bovis*													
JK115	1	1	16	16	4	8	1	1	4	>32	16	16	4
MF421524	1	2	16	16	4	4	1	1	8	8	16	16	8
WP2	1	2	8	8	4	4	1	1	8	>32	2	16	0.25
*P. ciferrii*													
WP3	1	2	8	8	4	4	1	1	4	>32	16	16	1
SAG 2063	1	2	8	8	4	4	1	1	4	>32	16	16	0.5
*P. blaschkeae*													
SAG 2064	1	4	4	8	8	8	2	2	2	>32	16	>32	0.25
*P. cutis*													
DSM 22084	1	2	4	4	4	8	1	4	0.5	>32	16	16	0.25
*P. miyajii*													
IFM 53848T	1	2	4	8	4	8	0.5	1	0.5	>32	16	16	0.5
*Candida* fungi													
*C. albicans*													
ATCC 10231	0.5	1	16	>32	4	4	1	1	1	>32	16	16	2
*C. guilliermondii*													
JK116	0.5	2	8	8	4	4	1	1	1	>32	8	8	0.25
Green algae													
*Auxenochlorella pyrenoidosa*													
JK171	4	>32	8	>32	8	>32	4	16	2	4	16	>32	16

^*a*^ All values in µg/mL.

The susceptibility profiles of *Candida* yeasts were similar to those of *Prototheca*. In contrast, the closely related photosynthetic alga *Auxenochlorella* displayed a markedly different susceptibility pattern (Table 1), highlighting the unique physiology of *Prototheca*.

**Fig 2:**
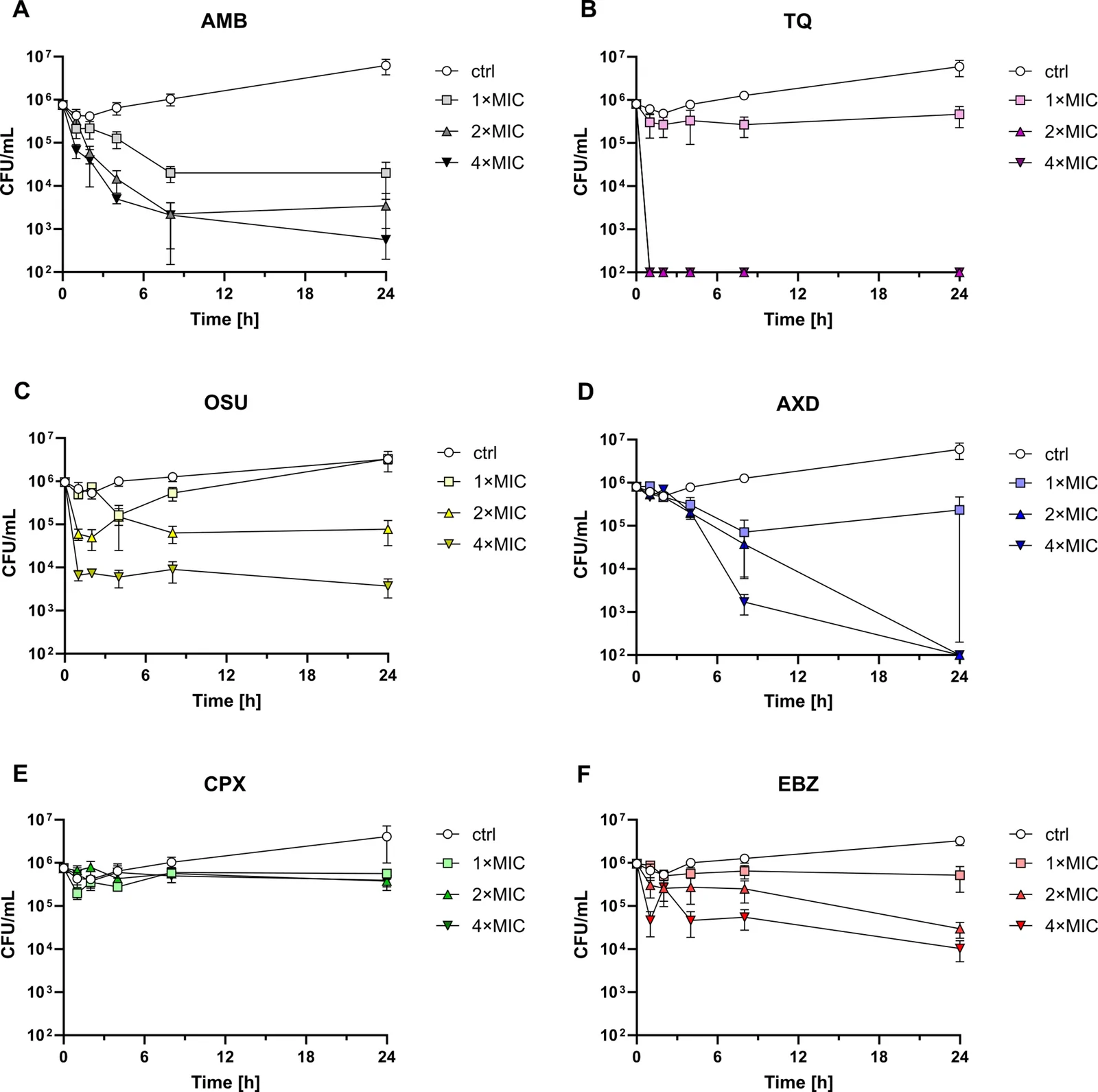
Selected compounds effectively kill *P. wickerhamii* CCUG 50031 when added at 1× MIC or higher concentrations, as evident by the time-kill analysis for (**A**) amphotericin B, (**B**) tafenoquine, (**C**) OSU-03012, (**D**) alexidine, (**E**) ciclopirox, and (**F**) eberconazole. Detection limit: 10^2^ CFU/mL. Data are shown as means ± SEM; *n* = 3.

Further characterization of compounds’ activity was performed with *P. wickerhamii* CCUG 50031 as a model strain. When time-kill analysis was performed, nearly all compounds showed some degree of cidal activity (Fig. 2A through F). TQ acted especially rapidly, completely eliminating viable *Prototheca* within 1 h at 2× MIC (Fig. 2B). AXD also showed strong killing activity, sterilizing cultures at 2× MIC, although more slowly than TQ (Fig. 2D). CPX was the only compound completely lacking cidal activity, thus being exclusively algistatic (Fig. 2E).

**Fig 3:**
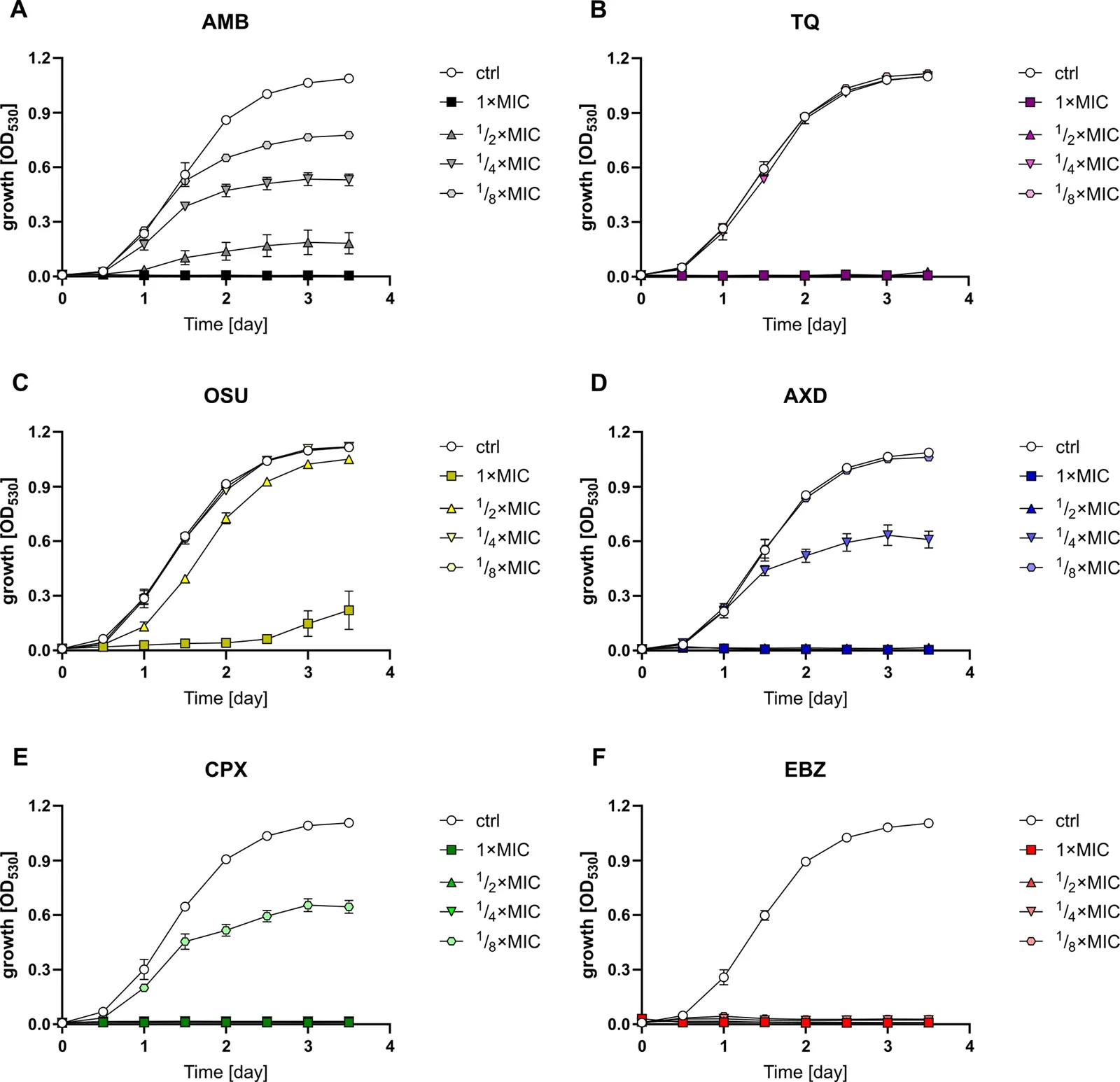
Selected compounds inhibit growth of *P. wickerhamii* CCUG 50031 even at sub-MIC concentrations, as analyzed spectrophotometrically over several days for (**A**) amphotericin B, (**B**) tafenoquine, (**C**) OSU-03012, (**D**) alexidine, (**E**) ciclopirox, and (**F**) eberconazole. Data are shown as means ± SEM; *n* = 4.

Long-term growth analyses revealed that, with the exception of OSU (Fig. 3C), all compounds retained growth-suppressive activity even at sub-MIC concentrations (Fig. 3). Marked inhibition of *Prototheca* growth was observed at concentrations as low as one-half, one-quarter, or even one-eighth of the MIC.

**Fig 4:**
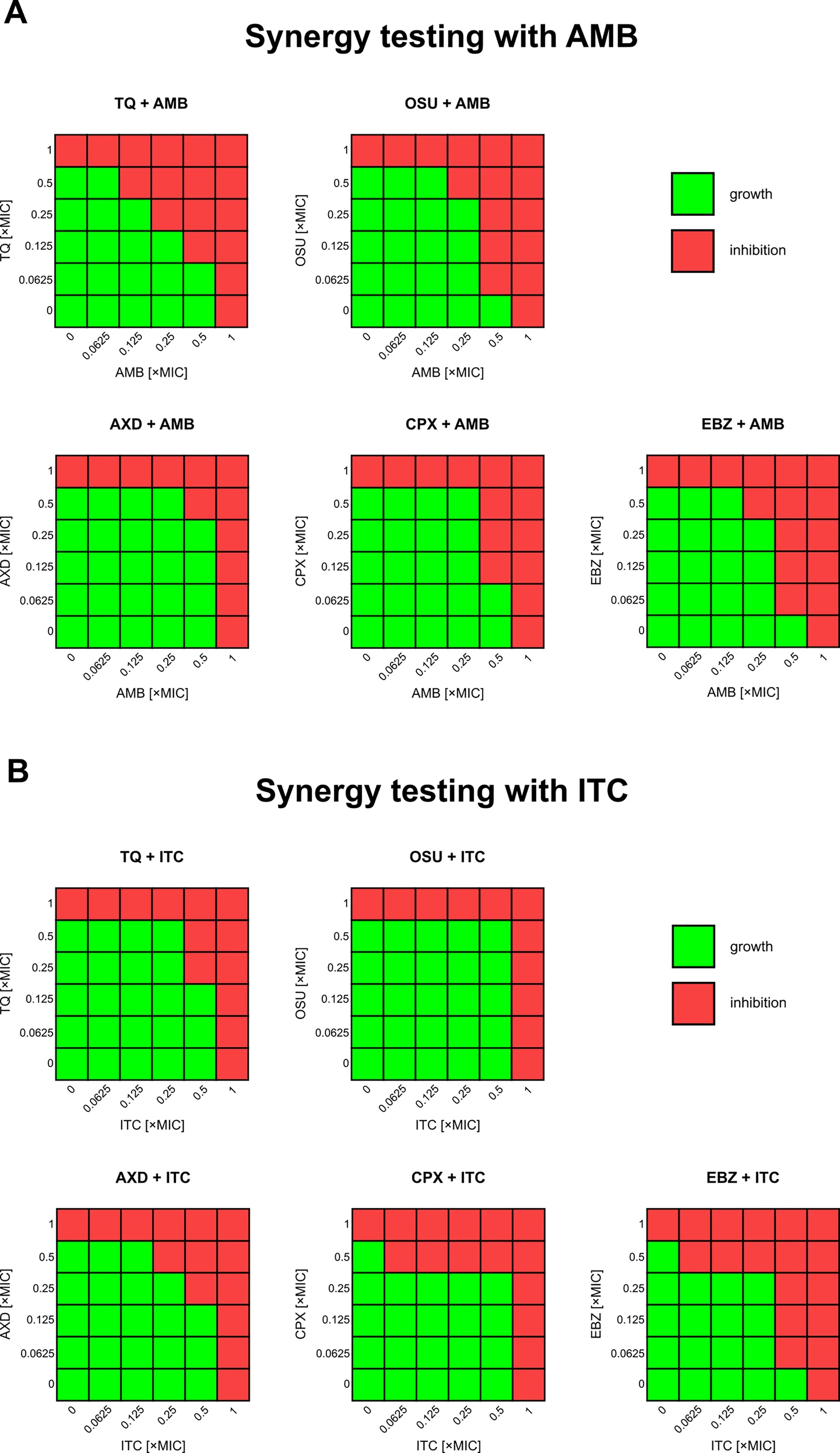
Identified compounds do not interact with the “traditional” antifungals used against *Prototheca*, AMB (**A**) and ITC (**B**). Tested compounds included TQ, OSU-03012, AXD, CPX, and EBZ, and *P. wickerhamii* CCUG 50031 was used for the tests. Based on the changes in concentrations of drugs needed to inhibit microbial growth alone or in combination, the fractional inhibitory concentration index was calculated, and 0.5 < ΣFIC < 1, observed for most of the compounds, was classified as “neither synergy nor antagonism” between the drugs. The only slight synergy was observed for TQ and AMB, where for both agents at 0.25× MIC, ΣFIC = 0.5.

Interactions between the newly identified compounds and the commonly used antifungals AMB and ITC were evaluated using the checkerboard method and fractional inhibitory concentration indices (ΣFICs) (26). Alone, the MICs for these drugs against *P. wickerhamii* CCUG 50031 were 1 µg/mL (AMB) and 8 µg/mL (ITC). Most of their combinations with the new compounds produced indifferent interactions (non-synergy, non-antagonism, as defined in more detail in Materials and Methods), with ΣFIC values >0.5 or ≤1 (Fig. 4A and B). The only indication of synergy was observed for the TQ + AMB combination, where both agents at 0.25× MIC showed ΣFIC = 0.5.

### Identified compounds are effective against *Prototheca* biofilms

**Fig 5:**
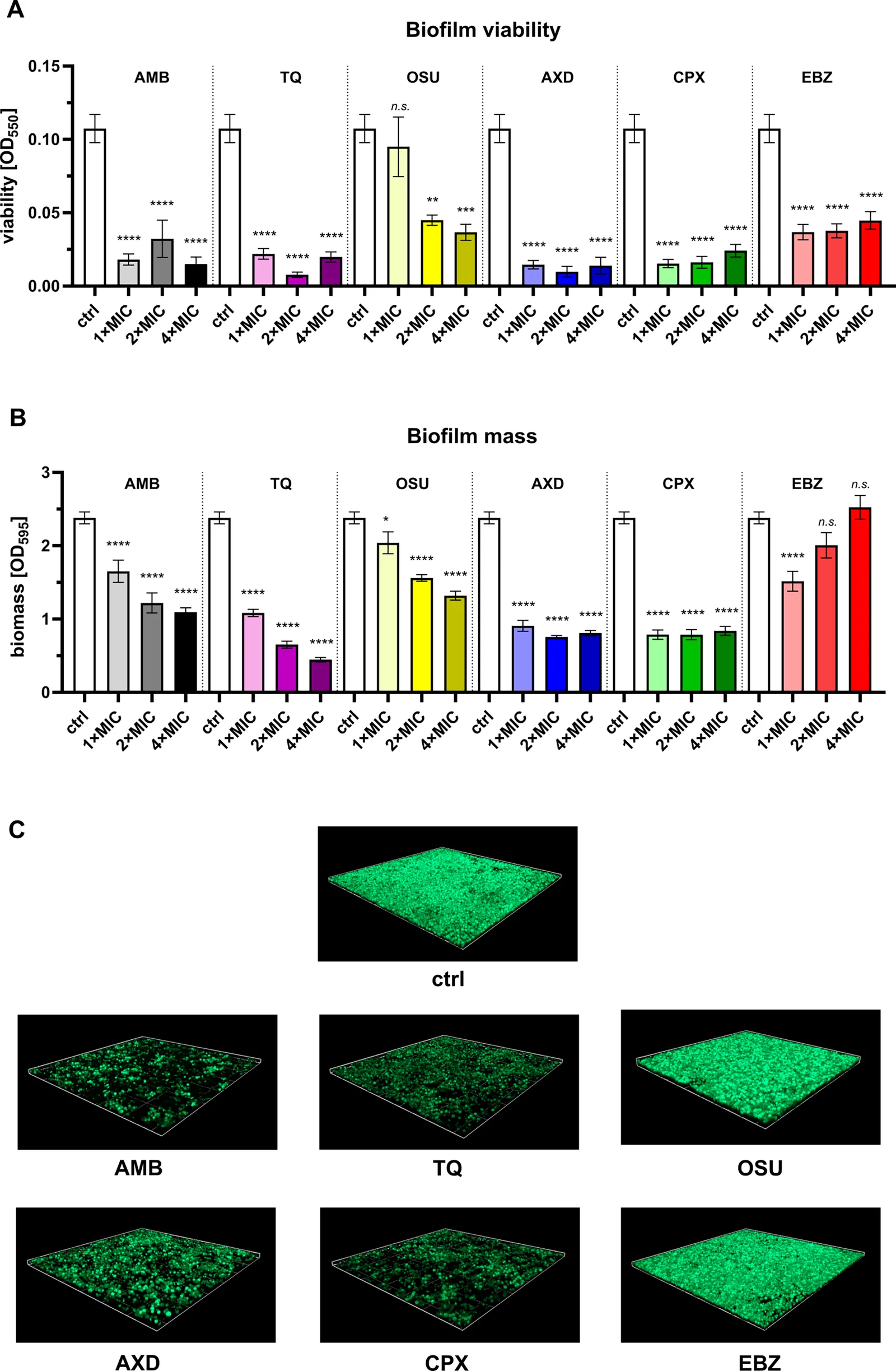
Identified compounds are active against pre-formed biofilms of *P. wickerhamii* CCUG 50031. (**A**) When added at different doses for 24 h to an already existing biofilm, the compounds reduced the viability of the biofilm as measured with a metabolism-sensitive MTT dye. (**B**) When added at different doses for 24 h to an already existing biofilm, the compounds reduced biofilm mass, as measured with crystal violet staining. (**C**) When added at 2× MIC for 24 h to an already existing biofilm, the compounds affected the biofilm three-dimensional structure, as measured with confocal laser scanning microscopy after staining with Syto9 fluorescent dye (image size: 600 × 600 × 20 µm). The tested compounds included AMB, TQ, OSU-03012, AXD, CPX, and EBZ. Statistical significances were determined by ANOVA with Dunnett’s post-test; ****P < 0.0001; ***P < 0.001; **P < 0.01; *P < 0.05; n.s., not significant. All data are shown as means ± SEM; *n* = 6 (compounds), *n* = 12 (ctrl), or as representative images.

*Prototheca* biofilms formed on artificial surfaces or host tissues display increased resistance to antimicrobials (13, 27). Consistent with this, preformed biofilms of *P. wickerhamii* CCUG 50031 were less susceptible to treatment than planktonic cells. When biofilm viability was measured with MTT staining (detecting metabolizing cells) after 24 h of compound exposure, substantial but incomplete reductions in biofilm viability were observed (Fig. 5A). Depending on the compound, biofilm viability decreased by 60% to 90%. Except for OSU, most compounds did not show a clear dose-dependent effect, suggesting that maximal activity was already reached at 1× MIC. This may reflect the presence of a small persister-like fraction of biofilm-associated *Prototheca* cells that remains viable despite antimicrobial treatment.

Analysis of total biofilm biomass (live and dead cells, and the extracellular matrix) using crystal violet staining revealed a more complex pattern (Fig. 5B). In contrast to viability measurements, biofilm biomass reduction was generally dose-dependent, suggesting that biofilm structure can persist even after substantial killing of embedded cells and requires higher drug concentrations for physical disruption. OSU again showed relatively weak activity, whereas EBZ paradoxically increased total biofilm biomass despite effectively reducing biofilm viability. This suggests that EBZ may stimulate the production of extracellular biofilm matrix. Similar effects were observed with confocal laser scanning microscopy: AMB, TQ, AXD, and CPX at 2× MIC disrupted biofilm structure and reduced surface coverage, while OSU and EBZ induced comparatively minor structural changes (Fig. 5C).

Although none of the tested compounds completely eradicated established *Prototheca* biofilms, the observed reductions in viability and biomass suggest that they may still substantially weaken biofilm integrity, allowing the host’s immune system to deal with its remains.

### Identified compounds have multiple mechanisms of action

A series of functional assays was performed on *P. wickerhamii* CCUG 50031 to characterize the mechanisms of action of the compounds.

**Fig 6:**
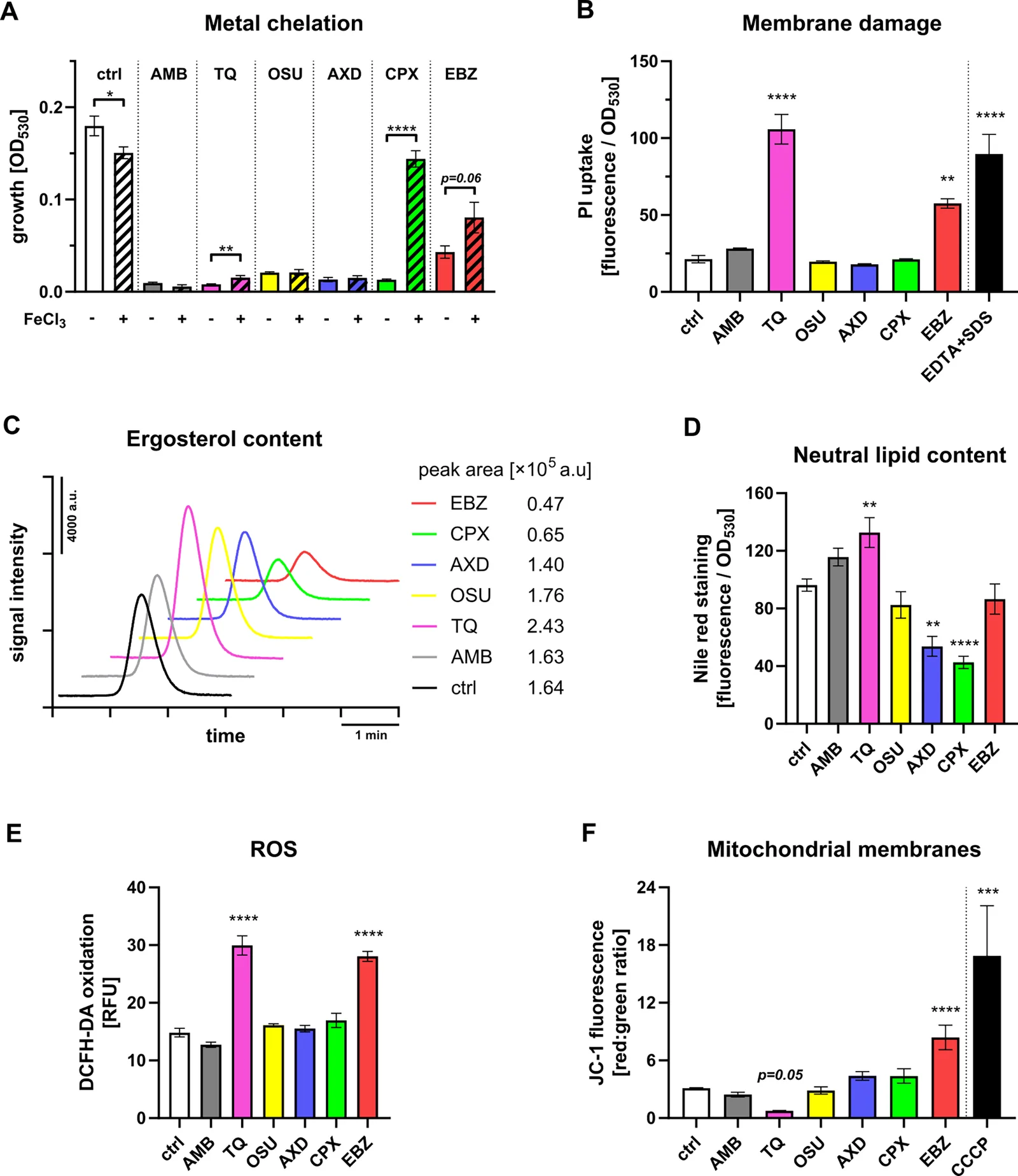
Identified compounds exert their anti-*Prototheca* activity through a variety of mechanisms. (**A**) To test for the compounds’ metal chelating activity, excess FeCl_3_ was added to algae with compounds at 1× MIC, and restoration of growth was assayed spectrophotometrically after 24 h. (**B**) To assess membrane damage caused by the compounds added at 1× MIC for 1 h, uptake of the membrane-impermeable propidium iodide (PI) was measured. (**C**) Loss of cellular ergosterol was quantified with chromatography in an equal number of cells grown with the compounds at 0.5× MIC overnight. For better visualization, chromatograms showing the ergosterol peaks for each compound are spread apart, each having a different time of initiation (*x*-axis) and baseline for signal intensity (*y*-axis). The amount of ergosterol is indicated by the area of the peak above its respective baseline (baseline being the flat part of the chromatogram on each side of the peak). (**D**) Changes in cellular neutral lipid levels were quantified through staining with Nile Red of the cells grown with the compounds at 0.5× MIC overnight. (**E**) Induction of reactive oxygen species (ROS) in cells exposed to the compounds at 0.5× MIC for 1 h was measured through fluorescence of a ROS-sensitive probe DCFH-DA. (**F**) Damage to mitochondria in cells exposed to the compounds at 1× MIC for 1 h was measured with fluorescent JC-1 staining sensitive to mitochondrial membrane potential. All experiments were performed with *P. wickerhamii* CCUG 50031. The tested compounds included AMB, TQ, OSU-03012, AXD, CPX, and EBZ. Statistical significances were determined by ANOVA with Dunnett’s post-test; ****P < 0.0001; ***P < 0.001; **P < 0.01; *P < 0.05. Data are shown as means ± SEM; *n* = 6 (**A**), *n* = 4 (**B and F**), *n* = 7 (**D**), *n* = 8 (**E**).

Addition of excess iron to culture medium with compounds at 1× MIC completely reversed the growth-inhibitory effect of CPX, indicating that iron chelation is central to its mechanism of action (Fig. 6A). A modest increase in growth following iron supplementation was also observed for TQ and EBZ, suggesting either their weak metal-chelating activity or, more likely, their affecting some iron-dependent metabolic pathways.

Membrane integrity was tested after 1 h of exposure to 1× MIC through uptake of the membrane-impermeable propidium iodide. TQ- and EBZ-treated cells showed marked membrane damage comparable to that induced by the membrane-disrupting EDTA/sodium dodecyl sulfate (SDS) control (Fig. 6B). AMB also damaged cellular membranes, but this effect developed more slowly and became detectable only after 24 h of treatment (Fig. S1).

Similarly to fungi, *Prototheca* contains ergosterol in the cell membrane (2). Quantification of ergosterol content in cells exposed to 0.5× MIC revealed a pronounced 60%–70% decrease following treatment with CPX and EBZ (Fig. 6C). For EBZ, this likely reflects direct inhibition of ergosterol biosynthesis, consistent with the mechanism of azole antifungals. In contrast, the effect of CPX was indirect and resulted from depletion of iron required for sterol biosynthesis, as supplementation with excess iron completely restored ergosterol levels (Fig. S2). Curiously, TQ increased cellular ergosterol content by over 40%, suggesting some substantial alteration of lipid metabolism by TQ.

Cellular neutral lipids, which serve as energy storage in algae, were also affected by treatment. Significant depletion of neutral lipid stores was observed after exposure to AXD and CPX (Fig. 6D). In contrast, TQ increased neutral lipid levels, further supporting the possibility of broad metabolic reprogramming induced by this compound.

Intracellular ROS production was measured after 1 h of exposure using the redox-sensitive dye DCFH-DA (2′,7′-dichlorodihydrofluorescein diacetate). A significant increase in ROS levels was observed in TQ- and EBZ-treated cells (Fig. 6E). Because mitochondria are the primary source of ROS in eukaryotic cells, mitochondrial status was assessed using the JC-1 dye, which reports changes in mitochondrial membrane potential through shifts in fluorescence ratio (Fig. 6F). Treatment with carbonyl cyanide *m*-chlorophenyl hydrazone (CCCP), an ionophore commonly used to dissipate mitochondrial membrane potential, markedly altered JC-1 staining. Unexpectedly, however, the direction of the fluorescence shift suggested an increase rather than the decrease in membrane potential typically observed following CCCP treatment. This raises uncertainty as to whether JC-1 staining reliably reflects mitochondrial membrane damage in *Prototheca*, and complicates interpretation of the assay results. Nevertheless, both TQ and EBZ induced some changes in JC-1 fluorescence relative to control cells. Although the exact meaning of these changes is unclear, they might be consistent with some type of mitochondrial membrane perturbation and suggest a link between mitochondrial function and the elevated ROS production observed in TQ- or EBZ-treated *Prototheca*.

### Identified compounds induce profound ultrastructural changes in *Prototheca*

**Fig 7:**
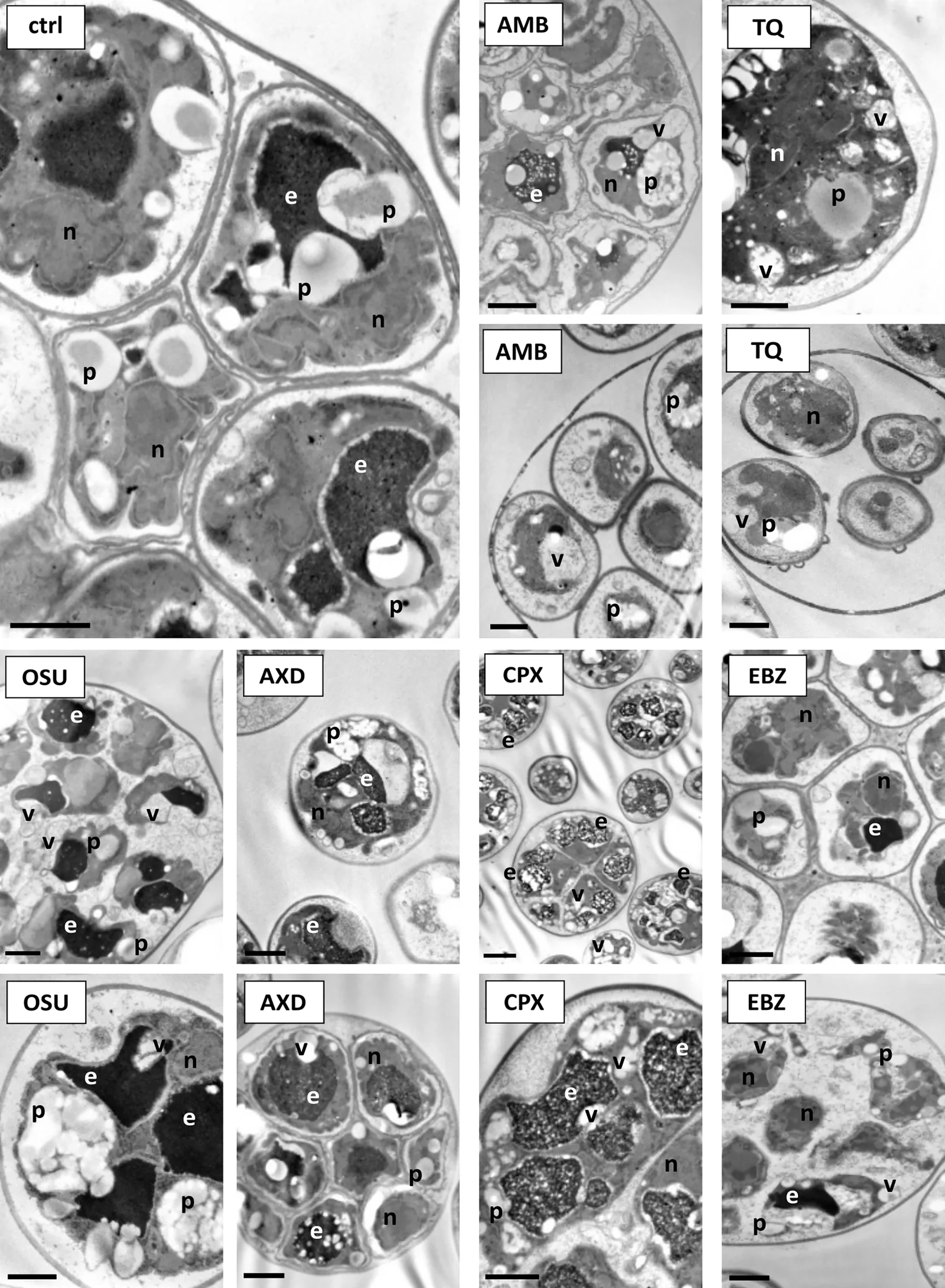
Identified compounds induce profound ultrastructural changes in *P. wickerhamii* CCUG 50031. Transmission electron microscopy analysis of the algae exposed for 1 h to the selected compounds revealed wide-ranging alterations caused by AMB, TQ, OSU-03012, AXD, CPX, and EBZ in comparison to control (ctrl) cells. Representative images are shown. Scale bar = 1 µm. Nuclei (n), electron-dense storage material (e), polysaccharides (p), vacuoles (v).

To further characterize the cellular effects of the identified compounds, the ultrastructure of *P. wickerhamii* CCUG 50031 exposed to 1× MIC for 1 h was examined with transmission electron microscopy (TEM) (Fig. 7).

AMB induced extensive structural deterioration consistent with necrotic cell death. Cell walls and cellular membranes were disrupted, while both polysaccharide and osmophilic (electron-dense, presumably lipid) storage materials in the cytoplasm were depleted. The cell nucleus was abnormal and contained a small amount of heterochromatin, and intracellular organelles displayed marked structural alterations. Organelles began changing their shape. These findings are consistent with previous reports describing ultrastructural changes after AMB exposure in *Prototheca* (28).

TQ induced pronounced apoptosis-like changes. Cells displayed highly condensed, electron-dense cytoplasm, extensive vacuolization, degeneration of intracellular organelles, depletion of both types of storage materials, membrane blebbing, and condensed chromatin.

In contrast, OSU caused comparatively mild ultrastructural changes, except for visible digestion of intracellular storage materials.

AXD treatment induced cytoplasmic shrinkage and vacuolization, accompanied by membrane disruption, abnormal mitochondrial morphology, and depletion of polysaccharide storage material, collectively suggesting the onset of apoptosis-like cell death.

CPX induced extensive apoptosis-like alterations. Cells showed depletion of storage materials, condensation and lobulation of nuclei, clustering of heterochromatin near the nuclear envelope, cell shrinkage, and disruption of plasma membranes, with leakage of cellular contents.

EBZ also triggered marked apoptosis-like changes, including nuclear lobulation, chromatin condensation, cytoplasmic shrinkage and condensation, membrane disruption, and formation of apoptotic-like bodies. Some digestion of the polysaccharide material was also visible.

Overall, TEM analysis demonstrated that while AMB primarily induced necrotic damage involving both intracellular structures and the cell wall, most newly identified compounds triggered apoptosis-like cell death without pronounced effects on the cell wall. A common feature of all treatments was depletion of intracellular storage materials, pointing to a previously underappreciated role of metabolic alterations in *Prototheca* response to antimicrobial agents. Notably, the degree of cellular damage observed by TEM was substantially greater than that detected by functional assays, indicating that ultrastructural analysis is highly sensitive for detecting cellular injury.

### Identified compounds are effective *in vivo* in a zebrafish infection model

To evaluate the translational potential of the identified compounds, a vertebrate infection model was used. Zebrafish larvae possess conserved innate immune cells (e.g., macrophages and neutrophils) and immune signaling pathways comparable to those of mammals and, owing to their optical transparency and availability of fluorescent reporter lines, enable real-time visualization of host–pathogen interactions *in vivo* (29). As a result, zebrafish larvae are increasingly used as a high-throughput, ethical, and reliable platform for *in vivo* antimicrobial evaluation (30).

**Fig 8:**
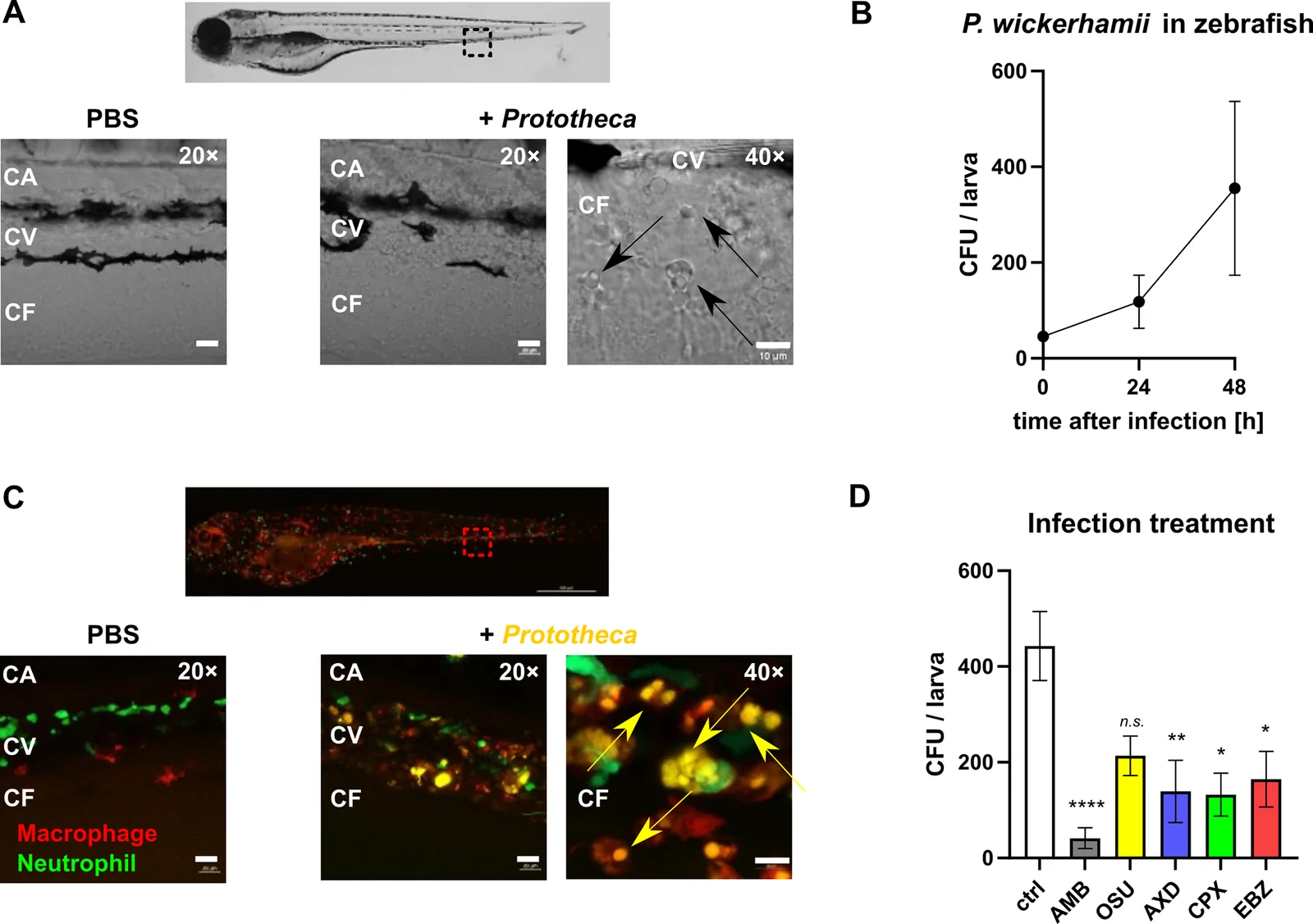
Selected compounds are effective for treatment of model *P. wickerhamii* CCUG 50031 infection in zebrafish larvae. (**A**) Systemically injected *Prototheca* (arrows) were found disseminated throughout the zebrafish body. Representative brightfield images of the larval tail (region indicated in the top panel by a square) after injection with phosphate-buffered saline (PBS) (left panel, 20× objective) or *Prototheca* (right panels, 20× and 40× objectives to better visualize individual *Prototheca* cells) 24 h post-infection (hpi). (**B**) Reflecting the microscopically observed systemic spread of the infection, the *Prototheca* CFU load was also increasing over the 48 hpi. (**C**) Infecting *Prototheca* (arrows) disseminated throughout the caudal fin tissues were attracting and interacting with fish neutrophils and macrophages (fluorescent imaging; *Prototheca*, yellow AF647; macrophage, red mCherry; neutrophil, green GFP; due to different camera optics architecture, the fluorescent image does not correspond to the exact same area as the brightfield image in panel **A**). (**D**) When injection of algae was followed 1–2 h later by a single injection treatment with 5× MIC of the selected compounds, they were able to greatly decrease the *Prototheca* burden in fish tissues at 24 hpi. The tested compounds included AMB, OSU-03012, AXD, CPX, and EBZ. Statistical significance was determined by ANOVA with Dunnett’s post-test, after log-transforming the data; ****P < 0.0001; **P < 0.01; *P < 0.05; n.s., not significant. All data shown as means ± SEM; *n* = 4–10 (**B**), *n* = 10–20 (**D**). CA, caudal artery; CV, caudal vein; CF, caudal fin. Scale bar: 10 µm.

After systemic injection of *P. wickerhamii* CCUG 50031, algal cells proliferated within the fish larvae and disseminated throughout the body (Fig. 8A and B). Macrophages and neutrophils were recruited to infection sites and interacted directly with *Prototheca* cells (Fig. 8C). Despite substantial microbial proliferation, infection caused only minimal larval mortality during the first 48 h.

Therapeutic efficacy was assessed by administering compounds 1–2 h after infection and quantifying microbial burden 24 h later. TQ proved toxic to zebrafish larvae and was therefore excluded from the experiments. All other compounds except OSU significantly reduced *Prototheca* burden after a single treatment dose (Fig. 8D). These findings demonstrate that AXD, CPX, and EBZ possess clear *in vivo* anti-*Prototheca* activity and highlight their translational potential for future therapeutic development.

## DISCUSSION

Treatment of *Prototheca* infections is challenging and often unsuccessful because of the limited availability of effective therapeutics. As the number of infections by these emerging pathogens is rising—in humans, dogs, and other animals—there is a pressing need to identify novel anti-*Prototheca* compounds and therapeutic targets. In this study, we screened the Pandemic Response Box compound collection and identified five compounds with promising anti-*Prototheca* activity: TQ, OSU, AXD, CPX, and EBZ. Previous screens of this compound library have successfully found candidate therapeutics targeting neglected fungal, bacterial, viral, and parasitic infections (31–37). In our case, all selected compounds showed broad activity against a range of clinically relevant *Prototheca* strains, some of them directly killing the algae, and others arresting its growth. The compounds’ mechanisms of action were characterized alongside AMB, which remains a commonly used treatment for protothecosis. Importantly, these compounds represented several distinct pharmacological mechanisms, demonstrating that chemical-library screening can uncover multiple exploitable vulnerabilities in organisms for which the therapeutic repertoire is limited.

### Analyzed compounds

AMB is thought to exert its antifungal activity through interaction with ergosterol in fungal membranes, either by forming membrane pores or by sequestering ergosterol from the membranes (38). Because *Prototheca* membranes also contain ergosterol, similar mechanisms have been proposed for these algae (2), although direct evidence has been lacking. Our results confirm that AMB induces membrane damage in *Prototheca*, albeit relatively slowly. In addition, we observed extensive intracellular damage and partial disruption of the cell wall, suggesting that AMB activity extends beyond the plasma membrane. Similar multifaceted effects have recently been described in yeast (39). Thus, the AMB-induced necrosis in *Prototheca* probably results from combined damage to cellular membranes, intracellular organelles, and cell walls.

TQ was originally introduced as an antimalarial drug (40), but has recently attracted attention for potential repurposing against trypanosomes, leishmania, bacteria, yeasts, and cancer cells (41–44). Its primary mechanism of action involves mitochondrial dysfunction and ROS overproduction (40). In *Prototheca*, TQ similarly targeted mitochondria and induced ROS accumulation. In addition, it caused membrane damage, altered lipid metabolism, and triggered apoptosis-like cell death. Comparable effects have previously been described in other organisms (41–44), suggesting that while mitochondria and oxidative stress may represent universal targets of TQ activity, additional downstream effects are likely organism-specific.

OSU, also known as AR-12, is a celecoxib derivative currently under clinical investigation as an anticancer agent (45), with additional attempts to repurpose it as an antibacterial, antiviral, antiparasitic, and antifungal drug. In cancer cells, OSU interferes with intracellular signaling and inhibits multiple enzymes, resulting in cell cycle arrest, endoplasmic reticulum stress, impaired autophagy, and apoptosis (45). In fungi, its activity has been linked more specifically to inhibition of acetyl coenzyme A synthetase, leading to mitochondrial dysfunction and impaired lipid synthesis (46). Compared with the pronounced effects reported in cancer and fungal cells, the effects of OSU in *Prototheca* appeared more subtle. Although the compound effectively inhibited growth and exerted algicidal activity, its precise mechanism of action remained unclear. The observed depletion of cellular storage materials suggests disruption of metabolic processes, consistent with possible inhibition of metabolic enzymes. However, no clear evidence of mitochondrial dysfunction or apoptosis-like death was detected, indicating that OSU may affect *Prototheca* through a distinct and potentially more specific mechanism.

AXD, a bisguanide antimicrobial, has broad antifungal activity (47), more limited antibacterial activity (48), and recently described anticancer properties (49). Although bisguanides were historically considered membrane-damaging agents (48), AXD appears to act through multiple mechanisms. Recent studies have linked AXD activity to mitochondrial dysfunction (49–51), limited membrane damage (50), ROS induction, and altered ergosterol synthesis (52), or apoptosis (51). In *Prototheca*, AXD displayed potent growth-inhibitory and cidal activity, although its mechanism differed somewhat from that reported in other systems. No ROS induction or major changes in ergosterol synthesis were detected. Instead, AXD treatment resulted in depletion of cellular storage materials and lipids, suggesting metabolic disruption. Ultrastructural analyses also revealed membrane and mitochondrial damage, although these effects were not clearly detected by functional assays. The most prominent phenotype induced by AXD was apoptosis-like cell death, suggesting that at least part of the observed ultrastructural damage may represent secondary consequences of the ongoing cell death process.

CPX is a broad-spectrum antifungal and antibacterial agent, whose primary mechanism of action is chelation of polyvalent metal cations (53). In *Prototheca*, CPX displayed strong anti-growth activity, and metal chelation appeared to be central to its mode of action. Although multiple downstream intracellular effects of CPX have been described previously (53, 54), these probably simply result from inhibition of metal-dependent enzymes. This was also evident in *Prototheca*, where ergosterol synthesis was strongly reduced by CPX treatment but restored after iron supplementation. Interestingly, CPX induced pronounced apoptosis-like ultrastructural changes despite exhibiting relatively limited killing activity. This apparent discrepancy suggests either that CPX preferentially affects actively dividing cells (leading to what appears as growth arrest of the whole population) or that at least some apoptosis-like changes remain reversible under certain conditions, as has been reported for apoptosis in animal systems (55). Notably, a single successful treatment of localized protothecosis using CPX in combination with antifungal terbinafine has previously been reported (56).

EBZ is a newly developed azole with broad activity against fungi (including azole-resistant strains) and gram-positive bacteria (57). Like other azoles, it primarily targets ergosterol synthesis and disrupts membrane integrity (57). In the present study, EBZ showed particularly strong activity against *Prototheca*, even exceeding its activity against fungi. Notably, EBZ proved to be much more potent than other antifungal azoles present among the screened compounds (Data S1). As expected, in *Prototheca,* EBZ inhibited ergosterol synthesis and induced membrane damage. However, it also disrupted mitochondrial function, increased ROS production, and triggered apoptosis-like cell death. Similar secondary effects involving mitochondria, oxidative stress, and apoptosis have previously been reported in fungi for some other azoles (58, 59).

### Targets and mechanisms of action

The identified compounds were active against a broad range of *Prototheca* strains and species. Most compounds induced substantial growth inhibition even at sub-MIC concentrations and, with the exception of CPX, also displayed cidal activity. None of the tested compounds completely eradicated established *Prototheca* biofilms, highlighting the pronounced resistance of biofilm-associated cells to treatment (27). Nevertheless, AMB, TQ, AXD, and CPX substantially disrupted biofilm structure, which may facilitate subsequent immune-mediated clearance. Such clearance would be impaired in immunocompromised individuals, emphasizing the need for developing compounds with improved anti-biofilm efficacy.

Important general insights into *Prototheca* biofilms also emerge from this study. The different effects on biofilm viability and its biomass and architecture demonstrate that killing cells and physically dismantling a biofilm are distinct processes that need to be investigated separately. The persistence, or even increase of biofilm mass despite reduced metabolic viability may indicate that extracellular matrix material remains stable after substantial cellular killing. Moreover, persistence of some viable metabolizing cells inside biofilm even after treatment with high doses of various compounds could represent distinct metabolically altered “persister-like” cell populations—a possibility that merits further investigation. Therefore, more research on *Prototheca* biofilm composition, structure, and metabolism will be needed to design treatments able to target all relevant aspects of biofilm infections.

One notable observation was the apparent lack of compounds specifically targeting the *Prototheca* cell wall. Apart from the limited wall-disrupting effects observed for AMB, none of the identified compounds appeared to act primarily through this structure. This may reflect the unusual composition and organization of *Prototheca* cell walls, which differ substantially from those of fungi and many other algae and appear highly resistant to enzymatic degradation (60). Although this resistance may complicate drug discovery, the distinctive nature of the *Prototheca* cell wall also makes it an attractive target for selective therapy. Further studies of cell wall composition, structure, and metabolism will therefore be essential.

Mitochondria emerged as another important intracellular target identified in this study. TQ and EBZ both disrupted mitochondrial function and induced ROS overproduction, suggesting that mitochondrial stress may represent a previously underappreciated vulnerability of *Prototheca*. Although ROS induction is not intentionally exploited by currently used antifungal therapies, mitochondrial dysfunction and oxidative stress have increasingly been recognized as secondary components of antifungal activity, particularly for azoles (58). These processes may therefore represent an overlooked common component of antifungal activity, worth further exploration in anti-*Prototheca* drug development. Moreover, several compounds intentionally targeting fungal mitochondria and ROS production are currently in preclinical development (61); therefore, the portfolio of such drugs available to repurpose against *Prototheca* is likely to expand in the near future.

Programmed cell death emerged as a common response to most tested compounds. *Prototheca* cells displayed multiple hallmarks of algal apoptosis-like death: organellar degeneration, cytoplasmic shrinkage and vacuolization, chromatin condensation, membrane blebbing, and formation of apoptotic-like bodies (62). Different compounds induced distinct combinations of these features, possibly reflecting activation of different programmed cell death pathways or different stages of the same process. Because apoptosis-like mechanisms in algae remain poorly understood (62–64), further studies of algal cell biology will be necessary to determine how these pathways can be therapeutically exploited. Notably, similar approaches are already being explored in antifungal drug development (65).

Another common effect observed across several compounds was disruption of cellular metabolism, manifested by depletion of intracellular storage materials and decreased levels of neutral lipids. In some cases, these changes may simply reflect a generalized stress response to cellular damage. However, they may also represent direct effects of the tested compounds on metabolic enzymes and pathways. Metabolic dysregulation has not previously been considered as an anti-*Prototheca* strategy and therefore represents an interesting direction for future drug development. At present, however, knowledge of *Prototheca* metabolism remains limited, with available insights derived primarily from genomic and transcriptomic analyses (66–68). Further research is therefore needed in this area.

### Translational potential of the identified compounds

None of the studied compounds showed any antagonistic activity against AMB (most successfully used for treatment nowadays) or ITC (less effective, but safer alternative to AMB), which means they could hypothetically be added to existing therapies without compromising their efficacy. Considering their *in vitro* activity, MIC values relative to the clinically used AMB, and *in vivo* efficacy in the zebrafish infection model, AXD, CPX, and EBZ appear to possess the greatest translational potential. TQ and OSU also remain interesting candidates despite their somewhat higher MIC values and lack of successfully demonstrated efficacy in the *in vivo* model. Importantly, TQ is already clinically approved for systemic treatment of malaria (40), while OSU has shown favorable tolerability in human clinical trials (46). These properties make both compounds attractive candidates for further evaluation as adjunctive therapies for protothecosis (probably in combination with existing antifungals).

AXD, CPX, and EBZ, all of which demonstrated efficacy in an *in vivo* animal infection model, are already available, primarily as topical formulations. Although this limits their immediate application in systemic protothecosis, they may still prove useful for localized or superficial infections, either alone or in combination with established antifungal agents. Development of systemic formulations may further expand their therapeutic potential. Notably, liposomal formulations of CPX for systemic treatment are already under development (69), and similar strategies could potentially be applied to EBZ or AXD.

### Limitations

Several limitations should be considered when interpreting our findings. Although the compounds were tested against a diverse panel of *Prototheca* isolates, the mechanistic experiments were performed using only one strain, and therefore the extent to which the identified mechanisms are representative for all species remains unclear. The mechanistic experiments established cellular phenotypes associated with activity and offered plausible pathways but did not definitively identify direct molecular targets. This is particularly relevant for mitochondrial dysfunction and apoptosis-like cell death, which may represent primary drug effects or secondary consequences of cellular stress. The zebrafish experiments provided proof of concept for *in vivo* activity, but mammal experiments, with models that mimic a realistic clinical treatment of protothecosis, will be needed to further confirm the translational potential of the compounds. Finally, while the initial screen clearly succeeded in identifying some potent anti-*Prototheca* compounds, it also has its limitations: only a single dose was used, and the screen was performed only twice, with results not always agreeing 100% between the repeats. It is therefore not an exhaustive assessment of the library, and future, more focused or differently designed screens of the same library might yield additional findings. Similarly, other limitations of this study will hopefully be addressed by future research, focused on precise drug target identification, exploration of structure-activity relationships, ruling out resistance development, and employing improved translational models.

### Summary

In summary, this study identified five compounds with previously unrecognized anti-*Prototheca* activity and characterized their mechanisms of action (Table 2). AXD, CPX, and EBZ showed particularly strong translational potential, while TQ and OSU may represent promising adjunctive candidates for systemic therapy. Beyond identifying candidate therapeutics, our findings highlight several biological processes that may represent exploitable vulnerabilities in *Prototheca*. These include targeting cell wall and membrane integrity, damaging mitochondria and causing oxidative stress, altering metabolism, and inducing apoptosis. At the same time, the study underscores how limited current knowledge of *Prototheca* biology remains. Our findings therefore not only expand the repertoire of potential anti-*Prototheca* therapeutics but also identify several key areas requiring further investigation into this unusual group of emerging microbial pathogens.

**TABLE 2:** Summary of anti-*Prototheca* properties of the tested compounds: AMB, TQ, OSU-03012, AXD, CPX, and EBZ^*a*^

Compound	*In vitro* effects	Biofilm eradication	Mechanism of action	Effective *In vivo*
AMB	Growth inhibition, strongly cidal	Moderate	Membrane damage; cell wall damage; cytoplasmic damage	Yes
TQ	Growth inhibition, strongly cidal	Moderate	Membrane damage; mitochondrial damage and ROS induction; metabolic changes; apoptosis induction	N.a.
OSU	Growth inhibition, cidal	Weak	Metabolic changes	No
AXD	Growth inhibition, cidal	Moderate	Metabolic changes; apoptosis induction	Yes
CPX	Growth inhibition	Moderate	Metal chelation; apoptosis induction	Yes
EBZ	Growth inhibition, cidal	Weak	Ergosterol synthesis inhibition; membrane damage; mitochondrial damage and ROS induction; apoptosis induction	Yes

^*a*^ N.a., not assessed.

## MATERIALS AND METHODS

### Microorganisms, growth conditions, and chemicals

Microbial strains are listed in Table 3. Species identity of *Prototheca* isolates was confirmed by partial *cytb* sequencing (70). *Prototheca* and *Candida* were grown overnight on Sabouraud dextrose agar (SDA; Millipore), and *Auxenochlorella* was grown for 7 days on modified Sager-Granick medium with 0.1% sodium acetate and 1.5% agar (71). All incubations were performed at 37°C, except for *Candida* and *P. cutis* (30°C), and *Auxenochlorella* (22°C, white light 40 µmol photons m^−2^ s^−1^).

**TABLE 3:** Microbial strains of *Prototheca*, *Candida,* and *Auxenochlorella pyrenoidosa* (formerly *Chlorella pyrenoidosa*) used in this study

Species	Strain	Source	Reference
*P. wickerhamii*	CCUG 50031	Human, skin infection	(27)
	PWPL1	Human, neuroinfection	(72)
	WP5	Dog, skin infection	(7)
*P. bovis*	JK115	Human, systemic infection	(73)
	MF421524	Human, peritonitis	(74)
	WP2	Dog, systemic infection	(7)
*P. ciferrii*	WP3	Dog, intestinal infection	(7)
	SAG 2063	Cattle, manure	(75)
*P. blaschkeae*	SAG 2064	Human, nail infection	(75)
*P. cutis*	DSM 22084	Human, skin infection	(76)
*P. miyajii*	IFM 53848T	Human, systemic infection	(77)
*C. albicans*	ATCC 10231	Human, lung infection	(78)
*C. guilliermondii*	JK116	Cattle, milk	–^*a*^
*A. pyrenoidosa*	JK171	Environmental	–

^*a*^ –, not available.

The tested compounds were AMB (BioShop #APH001), TQ succinate (Merck #SML0396), OSU-0301 (TargetMol #T2466), AXD (Merck #A8986), CPX (Merck #SML2011), and EBZ (TargetMol #T7824), all prepared as stock solutions in dimethyl sulfoxide (DMSO), which was included at matching concentrations as a control in all assays. In some experiments, ITC (Merck #419825) was also used. All other reagents were from Merck or Thermo Fisher Scientific.

### MIC and MCC determination

The MIC was determined by broth microdilution following European Committee on Antimicrobial Susceptibility Testing (EUCAST) yeast guidelines (E.Def 7.4). RPMI 1640 buffered with 3-(N-morpholino)-propanesulfonic acid and supplemented with 2% glucose (RPMI-2%G) was used as the medium. Inocula (0.5–2.5 × 10^5^ CFU/mL) were incubated in flat-bottom 96-well polystyrene plates (Sarstedt, 200 µL/well) with doubling drug dilutions, and growth was determined at 530 nm after incubation without agitation for 24 ± 2 h (or 7 days for *Auxenochlorella*). All wells contained a final 0.5% DMSO. MIC was defined as the lowest concentration causing ≥90% growth inhibition relative to controls. The MCC was determined by CFU counting in the wells after MIC determination and defined as the lowest concentration causing a ≥99.9% decrease in inoculum viability (19). MIC and MCC values represent modal results from three independent experiments.

### Compound screening

The Pandemic Response Box (Medicines for Malaria Venture) containing 400 compounds (22) was screened at 10 µM against *P. wickerhamii* CCUG 50031 using the MIC assay conditions. Growth inhibition was assessed at 24 and 48 h, and the screen was performed on two separate occasions.

### Growth curves and time-kill curves

For growth curves and time-kill assays, *Prototheca* was incubated with shaking in RPMI-2%G (≈1 × 10^6^ CFU/mL) containing compounds, either in 96-well plates (200 µL/well, OD_530_ measured, for growth curves) or in 1.5 mL tubes (1 mL/tube, 100 µL samples plated on SDA and CFU counted, for time-kill curves).

### Synergy testing

Compound interaction with AMB and ITC against *Prototheca* was tested with a checkerboard method (26), with experimental conditions and MIC growth inhibition cutoffs (90% and 50% for AMB and ITC, respectively) following the EUCAST method. The fractional inhibitory concentration index was calculated [ΣFIC = (MIC of compound A in combination MIC of compound A alone) + (MIC of compound B in combination/MIC of compound B alone)] for all combinations of different doses of tested compounds with AMB or ITC. Synergy was defined as ΣFIC ≤ 0.5, indifference as 0.5 < ΣFIC ≤ 4, and antagonism as ΣFIC > 4 (26).

### Biofilm destruction assays

*Prototheca* biofilms were formed over a 72 h incubation after inoculation into 200 µL of RPMI-2%G in wells of a flat-bottom 96-well polystyrene plate (or into 300 µL in an 8-well ibiTreat µSlide, Ibidi, for biofilm microscopy) at ≈3 × 10^5^ CFU/mL. Afterward, the medium was replaced with fresh medium containing the test compounds and incubated for an additional 24 h. Then, the medium was aspirated, the wells were washed with PBS, biofilm viability was quantified with thiazolyl blue tetrazolium bromide (MTT) staining (79), biomass was measured with crystal violet staining (19), and biofilm structure was visualized after SYTO-9 staining with a Zeiss LSM 900 laser scanning confocal microscope using a Plan-Apochromat 20×/0.8 objective (19).

### Metal chelation assay

To assess the contribution of metal chelation to antimicrobial activity of examined compounds, *Prototheca* was grown as for MIC determination, at 1× MIC of the compounds, with or without 50 µM FeCl_3_ supplementation, and its growth was measured as OD_530_.

### Cell membrane integrity measurement

Membrane integrity was evaluated using PI uptake. *Prototheca* at OD_600_ = 0.5 in RPMI-2%G with 1× MIC of compounds was incubated with shaking for 1 h or 24 h. Afterward, the cells were pelleted, stained with 60 µM PI in PBS for 15 min in darkness at room temperature, resuspended in fresh PBS, and fluorescence (ex./em. 485/535 nm) was measured and normalized to OD_530_. 20 mM EDTA + 0.02% SDS served as a membrane-damaging control (80).

### Ergosterol quantification

*Prototheca* cells grown overnight with shaking in 50 mL of RPMI-2%G containing compounds at 0.5× MIC were normalized to equal OD_530_, pelleted, and subjected to ergosterol extraction through alkaline ethanol saponification followed by hexane extraction (81). Two hundred microliters of the obtained extracts was evaporated under nitrogen and subsequently resuspended in an 80:20 (vol/vol) methanol:acetonitrile mixture, vortexed, centrifuged (9,000 × *g*, 1 min), and 150 µL of the resulting supernatant was injected onto a reversed-phase high-performance liquid chromatography (RP-HPLC) system (loop volume 100 µL) to separate and analyze the ergosterol (82) in the following RP-HPLC system: Jasco LC-4000 (Jasco Corporation) with a C_18_ reversed-phase column (Tracer Excel 120 ODS-A, 5 µm, 25 cm × 0.4 cm); methanol:acetonitrile 80:20 (vol/vol) eluent; 1 mL/min flow rate; and absorbance detection in the 240 nm–300 nm range, with the chromatogram recorded at the selected 280 nm channel. The area of the peak corresponding to ergosterol was calculated for chromatogram from each sample.

### Neutral lipid content quantification

*Protothec*a grown in the same conditions as those applied for ergosterol analysis was stained with Nile Red as described (83), and its neutral lipid content was measured as fluorescence (ex./em. 530/570 nm) and normalized to OD_530_.

### ROS and mitochondrial membrane potential measurements

To measure the ROS induction in *Prototheca*, a redox-sensitive dye, DCFH-DA (Merck), was used, as described previously (19), after 1 h of exposure to compounds at 1× MIC at 37°C. Fluorescence was read at ex./em. 485/535 nm.

The mitochondrial membrane potential of *Prototheca* (≈10^6^ CFU) incubated with compounds at 1× MIC in RPMI-2%G for 1 h was measured using JC-1 dye (MitoProbe JC-1 Assay Kit, Invitrogen, added for an additional 30 min of incubation) through flow cytometry per the manufacturer’s instructions.

### Transmission electron microscopy of algae

*Prototheca* grown for 1 h with shaking in 50 mL of RPMI-2%G with compounds at 1× MIC was centrifuged and resuspended in 2.5% glutaraldehyde in 0.1 M phosphate buffer (pH 7.4) for fixation (48 h, 4°C). Afterward, the material was washed twice with 0.1 M phosphate buffer, post-fixed in 1% osmium tetroxide, and, after washing with 0.1 M phosphate buffer, it was dehydrated in ethanol series (50%, 70%, 90%, 96%, 100%) followed by acetone, and embedded in epoxy resin. After staining with uranyl acetate and lead citrate, ultra-thin sections (70 nm) were visualized with a Hitachi H500 transmission electron microscope at 75 kV.

### Zebrafish larval infection model

Zebrafish (*Danio rerio*) were reared at the Zebrafish Core Facility, Institute of Zoology and Biomedical Research, Jagiellonian University. Wild-type larvae strain ABxTL and transgenic lines *Tg*(*mpeg1::mCherryF*)^ump2^ and *Tg*(*mpx::GFP*)^i114^ were used (84, 85). Larvae were maintained according to standard protocols in E3 medium with 0.00005% methylene blue, at 31°C, up to 4 days post-fertilization (86).

For infections, *Prototheca*, grown overnight with shaking in yeast-peptone-dextrose broth, washed, and resuspended in PBS at OD_600_ = 8, was injected (10 nL/larvae) into the duct of Cuvier of tricaine-anaesthetized zebrafish larvae at 30 h post-fertilization (87). At selected time points, larvae were anesthetized with tricaine, homogenized in 100 µL of ice-cold PBS, and CFU quantified by plating on SDA with 50 µg/mL chloramphenicol (to prevent growth of the zebrafish natural microflora).

For therapeutic experiments, 1–2 h after infection, the zebrafish larvae were injected into the duct of Cuvier with 2 nL of compounds dissolved in 50% DMSO (or with 50% DMSO alone as a control), to achieve 5× MIC inside the larvae (assuming 0.25 µL average larval volume). CFU per larva was determined at 24 hpi.

For imaging, *Prototheca* stained with Alexa Fluor 647 NHS Ester (Thermo Fisher Scientific) (88) was injected into double transgenic *Tg*(*mpeg1::mCherryF*)^ump2^ × *Tg*(*mpx::GFP*)^i114^ larvae with fluorescent macrophages and neutrophils. At 24 hpi, larvae were anesthetized, immobilized in 1% low-melting-point agarose in E3 medium, and visualized with a Zeiss LSM 900 laser scanning confocal microscope with an Airyscan 2 detector using Plan-Apochromat 20×/0.8 or C-Apochromat 40×/NA 1.2W objectives.

### Statistics

All data were collected in a minimum of two independent experiments. Differences between groups were analyzed by ANOVA with Dunnett’s multiple comparison post-test, or by *t*-test (where appropriate), with two-tailed *P*-values. CFU values were normalized by log transformation before analysis. Prism 7 (GraphPad Software) was used for calculations.

## SUPPLEMENTAL MATERIAL

Data S1 msphere.00427-26-s0001.xlsx Complete results of the compound screening.

Supplemental Figures msphere.00427-26-s0002.pdf Fig. S1 and S2.

ASM does not own the copyrights to Supplemental Material that may be linked to, or accessed through, an article. The authors have granted ASM a non-exclusive, world-wide license to publish the Supplemental Material files. Please contact the corresponding author directly for reuse.
