# Supplementary material for: Discovery of novel anti-Prototheca compounds with diverse modes of action through screening of a chemical library
Source: mSphere. 2026 Oct 7;11(10):e00427-26. doi: 10.1128/msphere.00427-26 (PMC13711791; doi:10.1128/msphere.00427-26)
Supplement: Supplementary file 1 [file msphere.00427-26-s0002.pdf]

# Supplementary Figures

## Discovery of novel anti-*Prototheca* compounds with diverse modes of action through screening of a chemical library

Amir Aliramezani, Magdalena Widziolek, Łukasz Chajec, Gabriela A. Żyłka, Beatrycze Nowicka, Izabela Ciastoń, Patrizia Danesi, Tomasz Jagielski, Magdalena Rost-Roszkowska, Tomasz K. Prajsnar, Jakub M. Kwiecinski

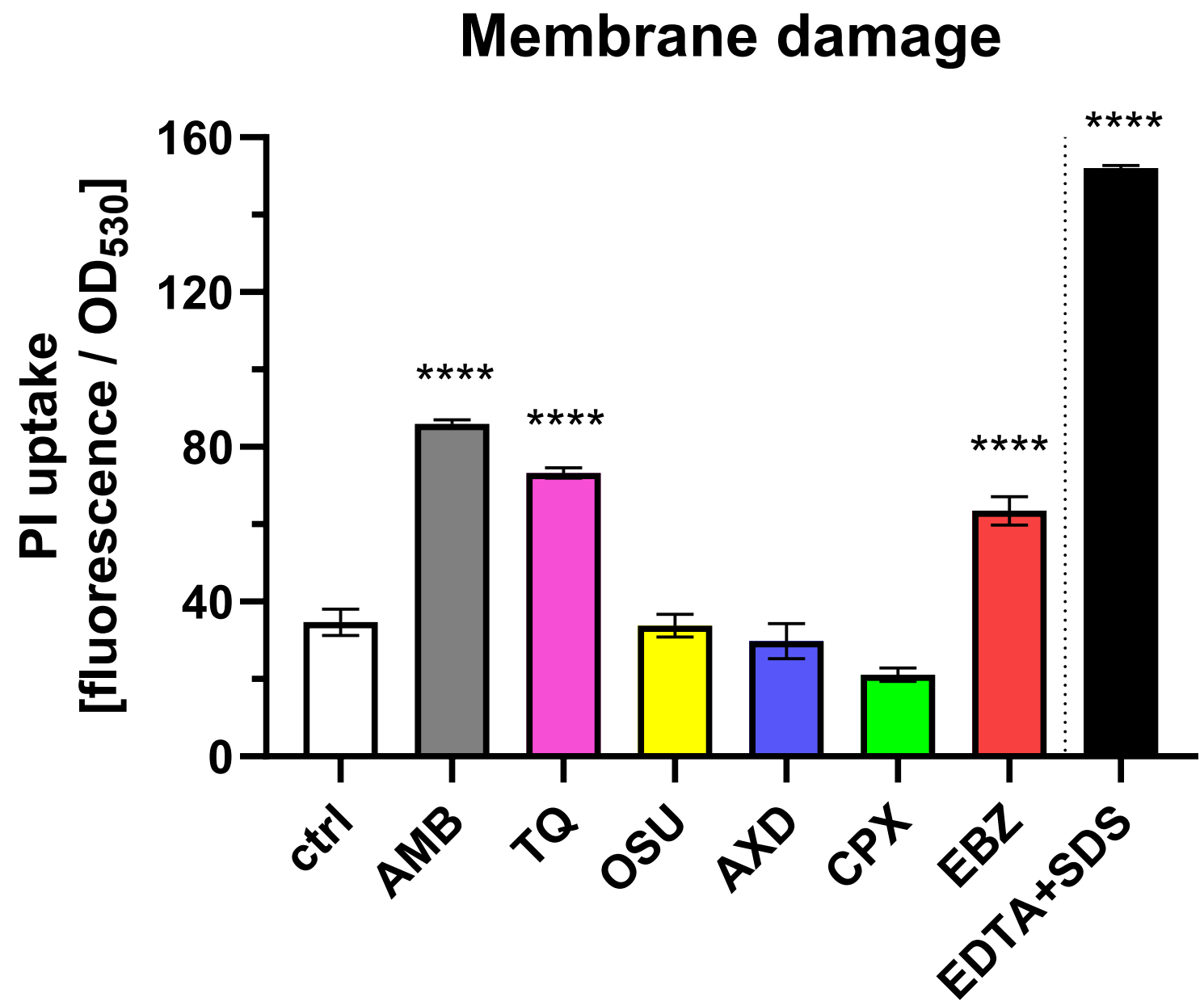


**Supplementary Figure 1.** To assess membrane damage caused by the compounds added at 1×MIC for 24 h, uptake of the membrane-impermeable propidium iodide (PI) by *P. wickerhamii* CCUG 50031 was measured. The tested compounds included amphotericin B (AMB), tafenoquine (TQ), OSU-03012 (OSU), alexidine (AXD), ciclopirox (CPX), and eberconazole (EBZ). Statistical significances were determined by the ANOVA with Dunnett's post-test; *****p*<0.0001. Data are shown as means ± SEM; n=4.

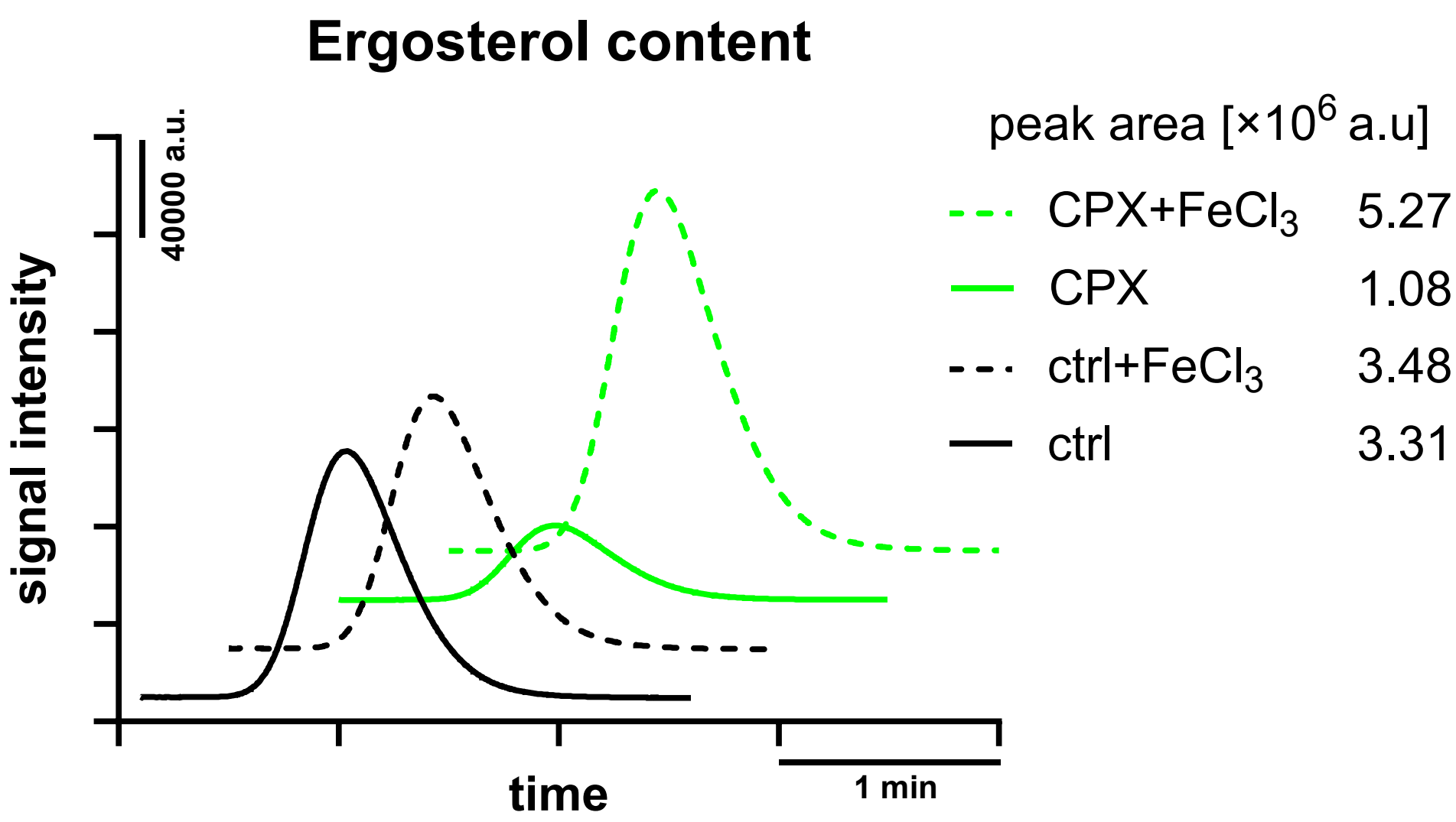


**Supplementary Figure 2.** Loss of cellular ergosterol was quantified with chromatography in *P. wickerhamii* CCUG 50031 cells grown with the ciclopirox (CPX) at 0.5×MIC overnight, with or without excess of $FeCl_3$ added.
